# *Hm*-MyD88 and *Hm*-SARM: Two key regulators of the neuroimmune system and neural repair in the medicinal leech

**DOI:** 10.1038/srep09624

**Published:** 2015-04-16

**Authors:** F. Rodet, A. Tasiemski, C. Boidin-Wichlacz, C. Van Camp, C. Vuillaume, C. Slomianny, M. Salzet

**Affiliations:** 1 Inserm U-1192, Laboratoire de Protéomique, Réponse Inflammatoire, Spectrométrie de Masse (PRISM), Université de Lille 1, Cité Scientifique, 59655 Villeneuve D'Ascq, France; 2 Inserm U-1003, Equipe labellisée par la Ligue Nationale contre le cancer, Laboratory of Excellence, Ion Channels Science and Therapeutics, Université Lille 1, Cité Scientifique, 59655 Villeneuve d'Ascq, France

## Abstract

Unlike mammals, the CNS of the medicinal leech can regenerate damaged neurites, thus restoring neural functions after lesion. We previously demonstrated that the injured leech nerve cord is able to mount an immune response promoting the regenerative processes. Indeed neurons and microglia express sensing receptors like *Hm*-TLR1, a leech TLR ortholog, associated with chemokine release in response to a septic challenge or lesion. To gain insights into the TLR signaling pathways involved during these neuroimmune responses, members of the MyD88 family were investigated. In the present study, we report the characterization of *Hm*-MyD88 and *Hm*-SARM. The expression of their encoding gene was strongly regulated in leech CNS not only upon immune challenge but also during CNS repair, suggesting their involvement in both processes. This work also showed for the first time that differentiated neurons of the CNS could respond to LPS through a MyD88-dependent signalling pathway, while in mammals, studies describing the direct effect of LPS on neurons and the outcomes of such treatment are scarce and controversial. In the present study, we established that this PAMP induced the relocalization of *Hm*-MyD88 in isolated neurons.

Innate immunity corresponds to the first line of defense common to all metazoans. To sense invading pathogens, animals align a panel of germline-encoded receptors called pattern recognition receptors (PRRs)^1^. Among them, the family of TLRs is the best characterized. TLRs are transmembrane proteins containing a Leucine-Rich Repeat (LRR) domain and a cytoplasmic Toll/Il1 receptor (TIR) domain. They detect and distinguish pathogen-associated molecular patterns (PAMPs) derived from various microbial pathogens including viruses, bacteria, protozoa and fungi^2^. The recognition of these PAMPs triggers the associated signaling pathways to activate downstream immune responses and eliminate invading pathogens^3^. Among the molecules recruited by activated TLRs are the members of the MyD88 family^4^. In mammals, it includes 5 adaptor proteins containing a TIR domain: myeloid differentiation factor 88 (MyD88), MyD88-adapter-like (Mal), TIR-domain-containing adaptor protein-inducing IFN beta (TRIF), TRIF-related adaptor molecule (TRAM) and sterile alpha and amardillo-motif-containing protein (SARM). All TLRs, except TLR3, recruit MyD88 to mediate innate immune signaling. MyD88 exhibits an N-terminal death domain (DD) and a C-terminal TIR domain. Upon stimulation of TLRs, MyD88 interacts with the cytosolic part of TLR through a homophilic interaction of the TIR domains. Its DD, in turn, associates with the DD of interleukin-1 receptor associated kinase (IRAK) to trigger downstream signaling cascades that lead to the activation of NF-κB^5^,^6^,^7^. The structure of MyD88 is extremely well conserved across evolution and its key role in immunity has been demonstrated in both bilaterian and non bilaterian species^8^,^9^,^10^,^11^,^12^,^13^,^14^. The second member of the MyD88 family particularly well conserved throughout the animal kingdom is SARM. Indeed, it is the only TIR domain-containing adaptor conserved from *C. elegans* to mammals^15^,^16^. SARM consists of two sterile-alpha motif (SAM) domains that are flanked by an N-terminal Armadillo motif (ARM) and a C-terminal TIR domain^17^. With this unique combination of three protein-protein interaction domains, SARM can bind a greater variety of interactants. Thus, SARM associates with mitochondria and/or microtubules in neurons, T-cells, Hela and kidney cells^18^,^19^,^20^,^21^,^22^. Moreover, in mammals^18^,^23^,^24^; protochordates^19^,^25^ and arthropods^16^,^26^, SARM also interacts with MyD88 and/or TRIF and/or TRAF6 to down-regulate TLR signaling. Noteworthy, SARM is the only negative regulator of the five TLR adaptor proteins. In contrast, SARM protects *C. elegans* against bacterial and fungal infection in a Tol-1-independent manner^15^,^27^ and restricts viral infections in mice brain^28^.

In the present study, we investigate such TLR adaptors in leech at the level of the CNS after infection or lesion. In fact, we previously established that the leech nerve cord is able to establish a specific neuroimmune response by discriminating microbial components^29^,^30^ or after lesion^29^,^31^,^32^. Indeed leech neurons and microglia express PRRs, and secrete antimicrobial peptides as well as chemokines in response to a septic challenge or injury^29^,^31^,^33^. A survey of the medicinal leech transcriptome and genome databases also reveals the presence of the main signaling molecules involved in the canonical TLR pathways^33^. We report here the characterization of two members of the MyD88 family: *Hm*-MyD88 and *Hm*-SARM. We demonstrated that their respective genes are tightly regulated during an immune challenge and CNS repair. A stimulation of leech isolated neurons with lipopolysaccharide (LPS) also triggered a redistribution of *Hm*-MyD88 at the cell surface. To the best of our knowledge, this clearly showed for the first time that differentiated neurons of the CNS could respond to LPS through a MyD88-dependent signaling pathway. Previously, we have also observed that stimulation of leech CNS with Muramyl dipeptide (MDP) induced the expression of the gene encoding *Hm*-TLR1^30^. We report here that this PAMP induced a relocalization of *Hm*-TLR1 and *Hm*-MyD88 in isolated neurons. A putative priming effect of MDP on TLR pathways and the possible existence of a cross talk between NLR sensor and TLRs in leech are discussed.

## Methods

### Microorganisms

The Gram-positive and Gram-negative bacteria, *Micrococcus nishinomiyaensis* and *Aeromonas hydrophila* respectively, were isolated from the natural environment of *Hirudo medicinalis* as previously described^34^. These bacterial colonies, which live in freshwater, were selected from agar plates under aerobic conditions at room temperature using a random isolation grid.

### Animals and treatments

Adult *H. medicinalis* were purchased from Ricarimpex (Bordeaux, France) and maintained in autoclaved pond water changed daily, for 1 week before starting any experimental procedure.

#### Microbial challenges *ex vivo*

Ten dissected nerve cords were collected per condition. Connectives between ganglia were injured in a standard manner using a pair of sterilized fine iridectomy scissors. Axotomized nerve cords were separately incubated in L-15 media containing different microbial components: 3 × 107 CFU/ml of heat-killed Gram positive (*M. nishinomiyaensis*) or negative (*A. hydrophila*) bacteria, 100 ng/ml of *E. coli* LPS (0111:B4 strain, Invivogen), 100 μg/ml of zymosan (Invivogen), 10 μg/ml of Muramyl dipeptide (MDP, Invivogen), 2 μg/ml of lipoteichoic acid (LTA) (Invivogen) or 10 μg/ml of Poly(I:C) (Invivogen) for different time (T0, t = 6 h) at room temperature. The supernatant of culture of cells infected with Vesicular stomatitis virus (VSV) for several days was used to stimulate the axotomized nerve cord *ex vivo*. Incubations without microbial components were performed in the same conditions as controls (H_2_O).

#### Regeneration process

Collected nerve cords were axotomized between each ganglion and cultured up to 8 days under sterile conditions. Ganglia were collected for RNA preparation or protein extraction at point 0 (T0), 6 h, 1day, 3days, 4 days and 8 days. Total RNA from 6 nerve cords were used in the RT-qPCR reactions. For western blot analysis, proteins were extracted from 3 isolated nerve cords.

### cDNA cloning

Partial sequences encoding *Hm*-MyD88 and *Hm*-SARM have been retrieved from the leech *H. medicinalis* nervous system EST database^33^. Full length cDNAs were generated by 5′-RACE using GeneRacer Core Kit (Invitrogen). Double stranded cDNAs from leech nervous systems were ligated to adaptors and these templates were used to PCR amplify 5′-RACE fragments using adaptor specific primers and gene-specific primers deduced from the initial fragment sequences. 5′-RACE-PCR were performed using 2.5 units of Platinum Taq polymerase (Invitrogen) in 1.5 mM of MgCl_2_. The cycling parameters were: 96°C/30sec; 5 cycles at 96°C/10sec and 72°C/4 min; 5 cycles at 96°C/10sec and 70°C/4 min; 25 cycles at 96°C/10sec and 68°C/4 min. All PCR products were cloned using pGemT-easy vector (according to the protocol provided by the manufacturer) and transformed into competent *Escherichia coli* JM 109 cells (Promega). Plasmids DNA were sequenced with a FM13/RM13 sequencing kit (Applied Biosystems) according to the manufacturer's instructions.

### Sequence and phylogenetic analyses

Translated sequences of *Hm*-MyD88 and *Hm*-SARM were used to search for conserved domains using the Simple Molecular Architecture Research Tool SMART™ 35 web server. Orthologous sequences from various vertebrates and invertebrates were retrieved from GenBank and used for the construction of phylogenetic trees using PhyML 3.0, www.phylogeny.fr^36^.

### Gene expression analysis

#### Gene expression in purified cells

The ganglia from 9 isolated nerve cords were carefully decapsulated by removing the collagen layer that envelops the nerve cord with microscissors. Neurons (>10 μm) and microglial cells (5 μm) were mechanically dissociated and resuspended in 200 μl of complete L-15 medium. The cells were then filtered through a 7 μm nylon mesh, as described previously^37^. According to their small size, purified microglial cells were collected with the eluate, while purified neurons were retained on the mesh. The latter were collected by gently scraping the inner face of the mesh in clean culture medium. RNA from purified cells were then extracted (Qiazol, Qiagen) and used for cDNA synthesis (Superscript II, Invitrogen) and PCR amplification (GoTaq, Promega). cDNA amplification was performed for 40 cycles at 95°C/30 s, 54°C (*Hm*-MyD88 and *Hm*-SARM) or 60°C (18 s reference gene)/2 min and 72°C/45 s (*Hm*-MyD88 and *Hm*-SARM) or 30 s (18S reference gene). These RT-PCR were performed with the following primers: *Hm*-MyD88 (forward primer: 5′-CTTCAAGATCCAAATGATGG-3′; reverse primer: 5′-AGTCTTCAGAGTAACAATCG-3′); *Hm*-SARM (forward primer: 5′-ACAACTTGCAAGTCTTCT-3′; reverse primer: 5′-GTAGTCATGTATCCATCTGATT-3′) the 18S reference gene (forward primer: 5′-TGCGGTTATTTCGATTGTCA-3′, reverse primer: 5′-AGACAAATCGCTCCACCAAC-3′).

#### Real time PCR quantification

20 axotomized nerve cords cultured at different times in the presence or absence of microbial components were used per condition. RNA extraction, cDNA synthesis, and real time PCR procedures were realized as already described^34^. The primers used for *Hm*-MyD88 (forward primer: 5′-GGAACTGGAAGACAAACAA-3′; reverse primer: 5′-GGGCTTAGGACGACAATGA-3′) and *Hm*-SARM (forward primer: 5′-TGCAACATCATTCCAGTCAT-3′; reverse primer: 5′-AATTGTTCCAGCTTGTCCAC-3′) quantification were designed with the Primer3 Input software^38^,^39^. The 18S was used as the reference gene (18S forward: 5′-TGCGGTTATTTCGATTGTCA-3′, 18S reverse: 5′-AGACAAATCGCTCCACCAAC-3′). Real Time reactions were conducted on a CFX96 qPCR system (BioRad) using a hot start, then 40 cycles at 94°C, 15 s; 56°C, 30 s; 72°C, 30 s, and a final extension step at 72°C for 3 min. Analysis of relative gene expression data was performed using the ΔΔCt method.

### Immunodetection of *Hm*-MyD88

#### Antisera

*Hm*-TLR1 protein was detected using the mouse antiserum as previously characterized by Ref. 40. The *Hm*-MyD88 antiserum was produced by Agrobio. The chemically synthesized immunogenic sequence (CIPDRDFLPGPPKYEAIT) was coupled to BSA and used for the immunization procedure of two rabbits.

#### Western blot analysis

For *Hm*-MyD88 characterization, 3 isolated nerve cords were disrupted in lysis buffer (6 M Urea, 2 M Thiourea, 40 mM Tris base) containing a cocktail of protease inhibitors (Sigma Fast Protease Inhibitor cocktail tablets EDTA free, Sigma-Aldrich). 20 μg of proteins were subjected to 14% SDS-PAGE and transferred to a nitrocellulose membrane (Protran, Whatman) by electroblotting. After transfer, the membrane was blocked for 1 h in PBS containing 0.1% Tween 20 and 5% skimmed milk and then probed either with the anti-*Hm*-MyD88 Ab or the anti-*Hm*-MyD88 Ab preincubated with the blocking peptide or the preimmune serum at a dilution of 1/10000 in blocking solution overnight at 4°C. After intensive washes with PBS/0.1% Tween20, the immunolabeled bands were detected using a peroxidase-conjugated anti-rabbit secondary Ab (1/50000 – Jackson ImmunoResearch; 60 min at room temperature). An ECL Western blotting kit (Amersham Biosciences) was used for chemoluminescence visualization with Kodak X-Omat AR film.

For protein expression analysis during CNS repair, 3 nerve cords were collected at each time point. CNS were then disrupted in PBS containing a cocktail of protease inhibitors (Sigma Fast Protease Inhibitor cocktail tablets EDTA free, Sigma-Aldrich). Proteins were precipitated with Acetone-10% TCA and resuspended in a solution containing 7 M Urea, 2 M Thiourea and 4% CHAPS. 20 μg (*Hm*-MyD88) or 30 μg (*Hm*-SARM) of proteins were subjected to western blot analysis as described above. The antibodies used were rabbit anti-*Hm*-MyD88 (1/10000) and rabbit anti-SARM (1 μg/mL, Novus Biologicals). To assess that an equal amount of proteins was loaded on gels, membranes were stripped with 0,2 M citric acid and reprobed with mouse anti-Actin (1/400, Thermo-Fisher).

#### Immunofluorescence

Neurons mechanically dissociated from nerve cords were fixed and centrifuged on slides. They were subjected to immunofluorescence procedure as follows. Cells were first blocked during 1 h with a blocking solution (PBS 1X, 1% Normal Donkey Serum, 0.01% Triton, 1% BSA) and then incubated overnight at 4°C either with rabbit anti-*Hm*-MyD88 or preimmune serum diluted 1/1200 in blocking solution. After intensive washes with PBS 1X, a biotin-conjugated donkey anti-rabbit (Jackson ImmunoResearch) diluted at 1/250 in blocking solution was applied for 1 h. After intensive washes with PBS 1X, the immune complex was revealed with a Rhodamine Red-X-Streptavidin (Jackson ImmunoResearch) at a concentration of 0.9 μg/mL. Slices were then mounted in glycergel (Dako) and examined using a confocal microscope (ZeissLSM510).

For immune challenge, isolated neurons were stimulated with 100 ng/mL lipopolysaccharide (LPS) or 10 μg/mL muramyl dipeptide (MDP) for 1 or 5 minutes respectively and double labeled with anti-*Hm*-MyD88 and anti-*Hm* TLR1 (1/100).

## Results

### Characterization of two MyD88 family members, *Hm*-MyD88 and *Hm*-SARM, in leech CNS

Analysis of the cDNA library of the leech CNS coupled to 5′ RACE-PCR allowed us to obtain the complete sequence of a molecule exhibiting the hallmarks of MyD88 (Supplementary Material 1): A death domain at the N-terminus and a C-terminal TIR domain. Thus, the leech molecule was named *Hm*-MyD88. This bioinformatics analysis revealed that *Hm*-MyD88 does not exhibit a signal peptide and is predicted as cytoplasmic. Interestingly, *Hm*-MyD88 also possesses the three highly conserved regions (Box 1, Box 2 and Box 3) present in most TIR domains. Moreover, the R-D-x-L-P-G motif, where x represents any amino acid, can be found in the Box 2. Indeed, in mammals, this motif is essential for the interaction of TLRs with MyD88^41^ and for MyD88 dimerization^7^. *Hm*-MyD88 also displays an additional C-terminal extension (CTE) as was described in the mussel *Mytilus galloprovincialis*^42^, the fruit fly *Drosophila melanogaster*^43^ and the mud crab *Scylla paramamosain*^44^. In the two arthropods, the CTE anchors MyD88 at the plasma membrane. Then, MyD88 recruits its activated Toll receptor and downstream cytosolic adaptors to trigger Toll signaling in response to an immune challenge^45^.

**Figure 1:**
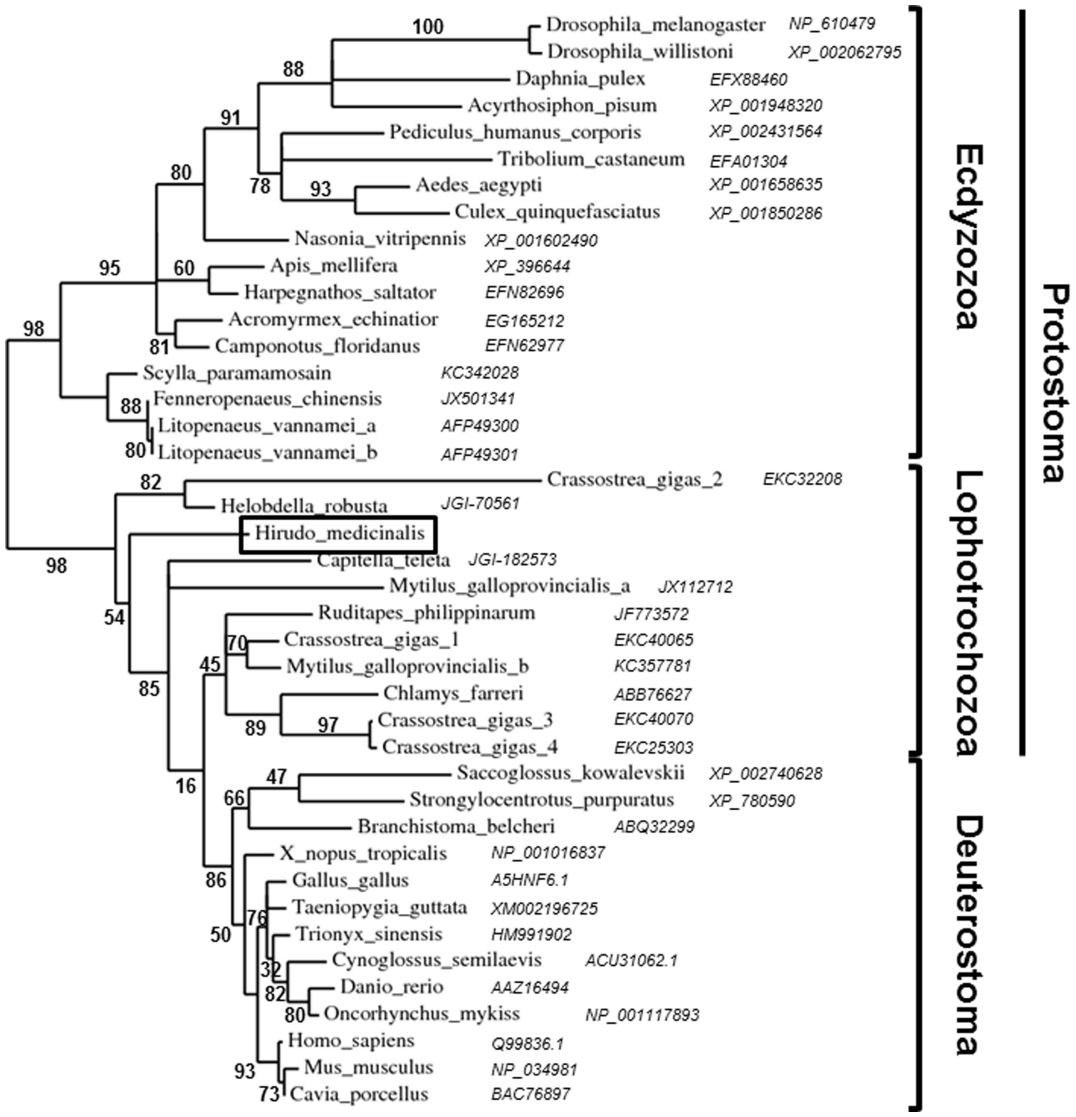
Phylogenetic representation of the relationship between *Hirudo medicinalis* MyD88 (*Hm-*MyD88), vertebrate MyD88 and invertebrate MyD88. The tree was built using the *phylogeny.fr* web server. Numbers correspond to the percentages of bootstrap values over 100 replicates.

**Figure 2:**
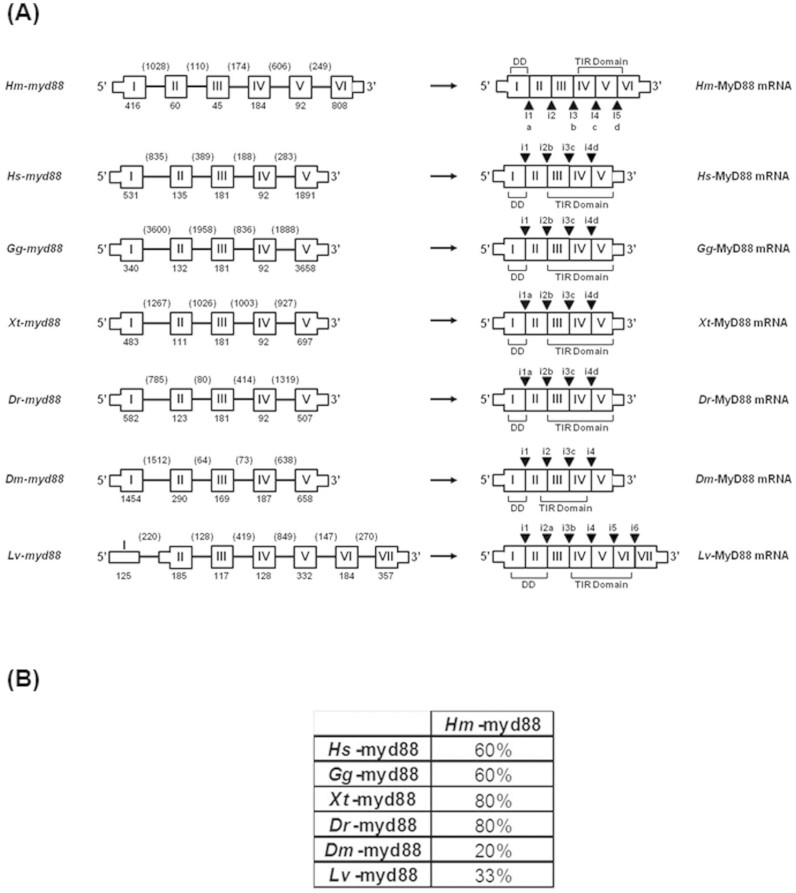
Comparison of the MyD88 gene structures in leech, vertebrates and ecdyzozoa. (A) The structural organization of *Hm*-*myd88* was deduced after the analysis of the leech genomic library. The exons are boxes marked I-VI; I-V or I-VII. Thick boxes represent the coding regions and thin boxes represent the non coding regions. The introns are given as solid lines. The numbers specify the length (bp) of the exons and introns. On the transcripts, black Δ marked i1-i5; i1-i4 or i1-6 indicate the position of the intron-exon junctions. Letters a, b, c and d point out which leech intron-exon junction is conserved in vertebrate or ecdyzozoa genes. For example, i2b in the human gene indicates that the position of the second intron occurs at the same position as the third intron in leech gene. (B) Percentage of conserved intron-exon junctions between leech gene and vertebrate or ecdyzozoa genes.

A search in the EMBL/Genbank databanks revealed 36%–18% identity and 53–37% similarity of *Hm*-MyD88 with vertebrate, protochordate and invertebrate MyD88s (Supplementary Material 1). Surprisingly, *Hm*-MyD88 shares higher sequence identities and similarities with deuterostomian orthologs than with protostomian MyD88s. Phylogenetic analysis was carried out to investigate the relationship between vertebrate and invertebrate MyD88s (Figure 1). The resulting tree exhibits three distinct groups: the deuterostomian group, the lophotrocozoan group and the ecdyzozoan group. Note that the lophotrocozoan group clusters closer to that of the deuterostomian group. In addition, this analysis showed that annelid MyD88s including *Hm*-MyD88 emerged early during evolution, before the divergence between Lophotrochozoa and Deuterostoma. To deal with this phylogenetic analysis in depth, the structural organization of the *Hm*-MyD88 gene was compared to those of the vertebrate and ecdyzozoa genes (Figure 2A). This revealed that *Hm-myd88* exhibits a different organization. It displays 6 exons and 5 introns while the vertebrate and Drosophila genes have 5 exons and 4 introns and the shrimp gene possesses 7 exons and 6 introns. However, 60 to 80% of exon-intron boundaries in *Hm-myd88* occur at the same position as those of vertebrate genes versus only 20 to 33% for the ecdyzozoa genes (Figures 2A, 2B and Supplementary data 3). This again proves the higher phylogenetic proximity of *Hm*-MyD88 with the deuterostomian orthologs.

**Figure 3:**
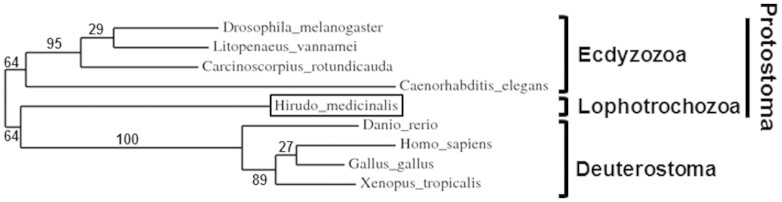
Phylogenetic representation of the relationship between *Hirudo medicinalis* SARM (*Hm-*SARM), vertebrate SARM and invertebrate SARM. The tree was built using the *phylogeny.fr* web server. Numbers correspond to the percentages of bootstrap values over 100 replicates. Accession numbers in EMBL/Genbank: *Hs*SARM: *Homo sapiens* (Q6SZW1); *Gg*SARM: *Gallus gallus* (A5HNF6.1); *Xt*SARM: *Xenopus tropicalis (*XM_002937143.2); *Dr*SARM: *Danio rerio* (B3DK97); *Dm*SARM: *Drosophila melanogaster* (Q6IDD9); *Lv*SARM: *Litopenaeus vannamei* (G8GV23); *Cr*SARM: *Carcinoscorpius rotundicauda* (A9X3T4); *Ce*TIR-1: *Caenorhabditis elegans* (Q86DA5).

**Figure 4:**
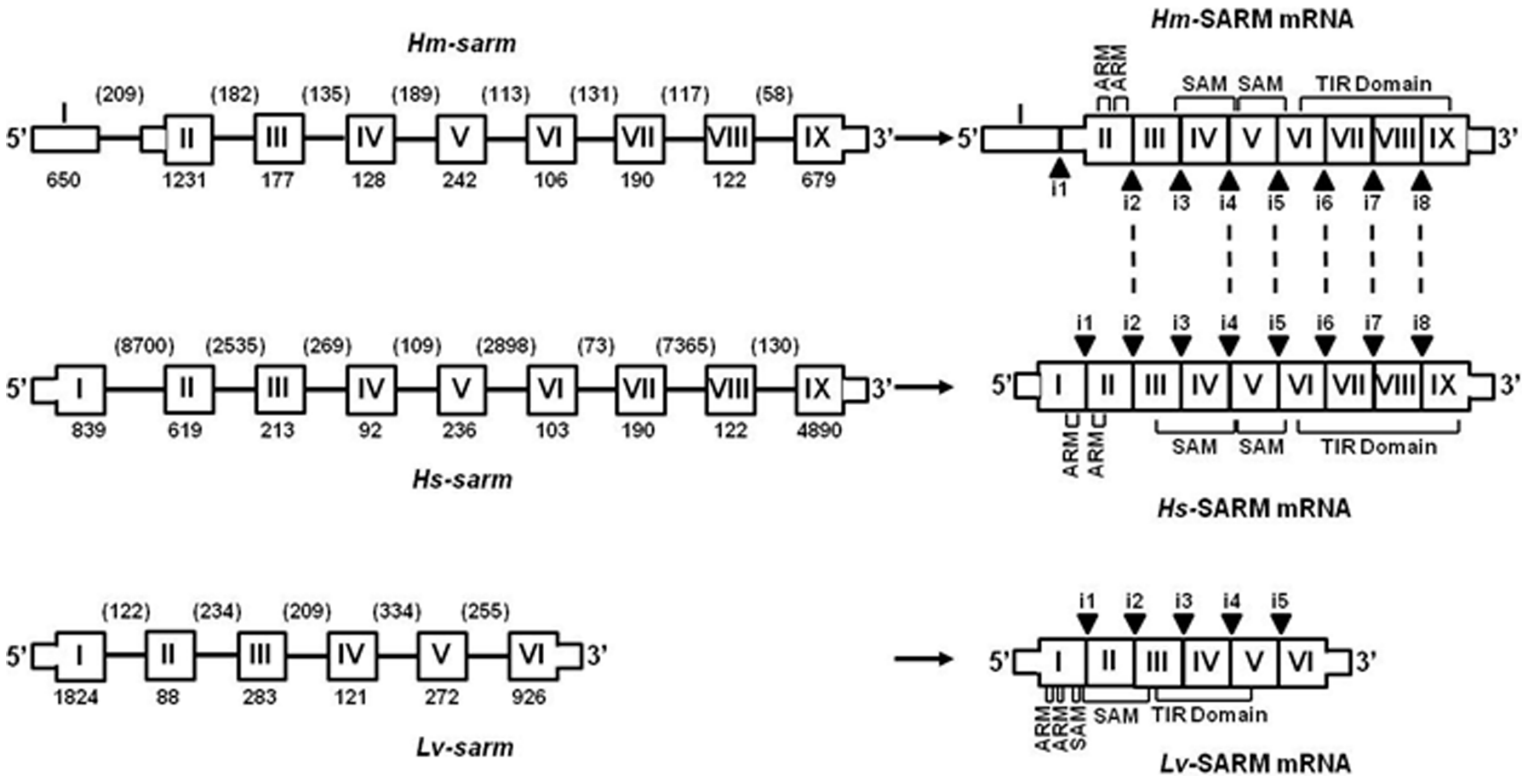
Comparison of the *sarm* gene structures in leech, human and shrimp. The structural organization of *Hm*-*sarm* was deduced after analysis of the leech genomic library. The exons are boxes marked I-IX or I-VI. Thick boxes represent the coding regions and thin boxes represent the non coding regions. The introns are given as solid lines. The numbers specify the length (bp) of the exons and introns. On the transcripts, black Δ marked i1-i9 or i1-i5, indicate the position of the intron-exon junctions. Dashed lines point out that the position of an intron-exon junction is the same in leech and human genes.

In parallel to the characterization of *Hm*-MyD88, a partial sequence orthologous to vertebrate and invertebrate SARM was retrieved from the cDNA library of the leech CNS. From this cDNA fragment, a complete sequence encoding *Hm*-SARM was cloned using 5′ RACE-PCR (Supplementary Material 2). It exhibits the hallmarks of SARM, that is, the two sterile-alpha motif (SAM) domains that are flanked by an N-terminal Armadillo motif (ARM) and a C-terminal TIR domain^17^. A search in the EMBL/Genbank databanks revealed 40%–23% identity and 55–34% similarity of *Hm*-Sarm with vertebrate and invertebrate SARM (Supplementary Material 2). Phylogenetic analysis was carried out to investigate the relationship between vertebrate and invertebrate SARM (Figure 3). The resulting tree exhibits three distinct groups as was described for *Hm*-MyD88: the deuterostomian group, the lophotrocozoan group and the ecdyzozoan group. As in *Hm*-MyD88, the lophotrocozoan group clusters closer to that of the deuterostomian group. Again, the hypothesis of a higher phylogenetic proximity between these two groups is supported by the study of the gene structure organizations (Figure 4). Indeed, *Hm-sarm* exhibits the same organization as in the human gene, *i.e.* 9 exons and 8 introns, while the shrimp gene displays 6 exons and 5 introns. Moroever, 6 of the 8 exon-intron boundaries in *Hm-sarm* and the human gene occur at the same position whereas none occurs in the shrimp gene (Figures 4 and Supplementary data 4).

### Expression of *Hm*-MyD88 and *Hm*-SARM molecules in leech CNS

**Figure 5:**
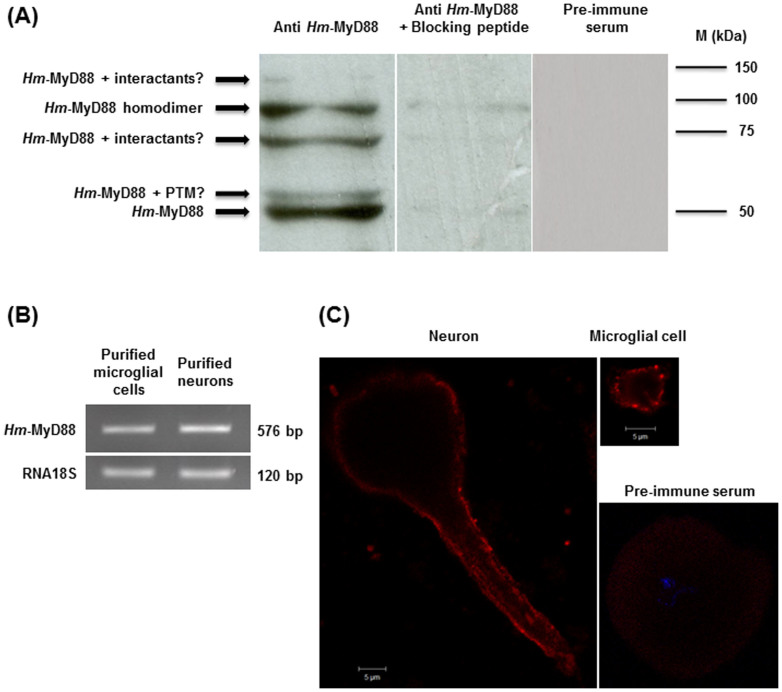
*Hm*-MyD88 is expressed in the central nervous system of the leech. (A) Western blot analysis of leech CNS extracts with anti-*Hm*-MyD88. To assess the specificity of anti-*Hm*-MyD88, two controls were performed: An incubation with *Hm*-MyD88 antibody pre-incubated with the blocking peptide and a reaction with the pre-immune serum. PTM: Post translational modifications. (B) Detection of *Hm*-MyD88 transcript by RT-PCR in leech purified neurons and microglial cells. (C) Cellular localization of *Hm*-MyD88 in neurons and microglia was determined by immunofluorescence and confocal microscopy analyses. No immunolabeling was observed when incubated isolated neurons with the pre-immune serum.

To continue the characterization of *Hm*-MyD88, a Western blot analysis of leech CNS extracts was performed using an anti-*Hm*-MyD88 (Figure 5A). To assess the specificity of *Hm*-MyD88 recognition, two controls were used (i) A reaction with the *Hm*-MyD88 antibody pre-incubated with the blocking peptide and (ii) a reaction with the pre-immune serum. Five specific bands were detected. The most intense band at 45,4 kDa is consistent with the predicted mass deduced from *Hm*-MyD88 cDNA. The upper band at 50 kDa may correspond to *Hm*-MyD88 bearing post translational modifications such as phosphorylations. Indeed, bioinformatics analysis revealed several potential sites of phosphorylation of serine, threonine and tyrosine residues. In mammals, MyD88 phosphorylation is crucial to allow its recruitment and activation^4^. Besides, mammalian MyD88 function as homodimers^7^. The presence of the R-D-x-L-P-G motif (Supplementary Material 1) and a band around 90 kDa suggest that *Hm*-MyD88 seems also to function as a dimer interface. The two other bands at 75 kDa and 150 kDa could correspond to *Hm*-MyD88 still bound to its interactants.

As shown by RT-PCR, *Hm*-MyD88 transcript was detected in purified neurons and microglial cells in basal conditions (Figure 5B). Cellular localization of *Hm*-MyD88 in the absence of infection was further investigated in neurons and microglia by immunofluorescence and confocal microscopy analyses (Figure 5C). In microglia, *Hm*-MyD88 was localized adjacent to the plasma membrane. In neurons, the protein was detected in the cell bodies and along the axons as aggregates in the vicinity of the cell surface. This is consistent with the presence of a CTE that could anchor *Hm*-MyD88 at the plasma membrane and it corroborates the results of the Western blot. In Hela cells, MyD88 associated with beta-actin^46^. Interestingly, some *Hm-MyD88* positive fibers were also revealed along the axons Thus, it may also interact with beta-actin. This suggests the antero-grade transport of this adapter at the cut-ends of the axons or its internalization.

**Figure 6:**
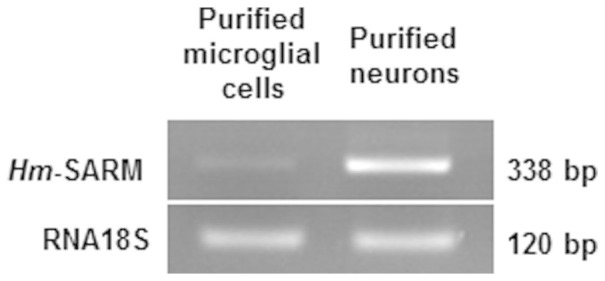
Detection of *Hm*-SARM transcript by RT-PCR in leech purified neurons and microglial cells.

The pattern of expression of *Hm-*SARM in leech CNS was also carried out by RT-PCR (Figure 6). Interestingly, SARM transcripts were strongly detected in neurons and were only slightly expressed in microglial cells. This is reminiscent of the situation found in mouse brain^20^.

### Modulation of *Hm*-myd88 and *Hm*-sarm gene expression upon microbial challenge of the leech CNS

**Figure 7:**
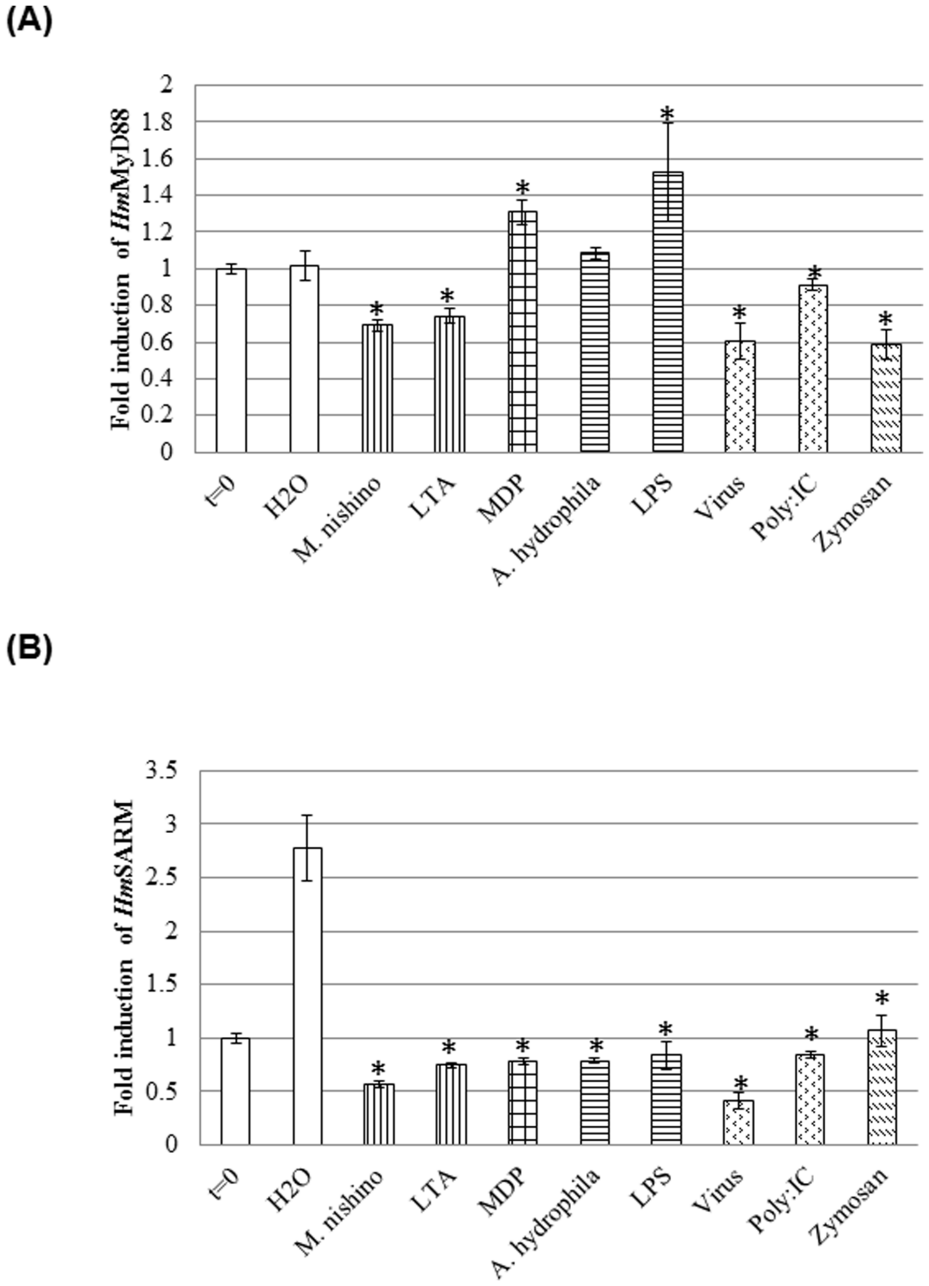
Modulation of expression upon experimental microbial challenges. The modulation of (A) *Hm*-MyD88 and (B) *Hm*-SARM gene expression were investigated in nerve cords incubated for 6 h with various microbial components (LTA: lipoteichoïc acid; MDP: muramyl dipeptide; LPS: lipopolysaccharides; zymosan), killed microorganisms (M.nishino: *Micrococcus nishinomiyaensis*; A. hydrophila: *Aeromonas hydrophila*; VSV: Vesicular stomatitis virus) or viral mimetic poly(I:C). Graphics represent the best results of two independent experiments that displayed similar variations; p-values from Student's T-tests were calculated versus the control treatment (H_2_O), based on experimental measurements performed in triplicate (*p < 0.05).

We previously showed that the leech nerve cord is able to discriminate microbial components present in its environment^29^. Indeed, the presence of various components that derive from or mimic the presence of different microorganisms *e.g.* bacteria, fungi or viruses, modulated the expression of the genes encoding various PRRs, AMPs and chemokines^29^,^30^,^31^. To assess if such experimental infections also modulate the gene expression patterns of *Hm*-MyD88 and *Hm*-SARM, quantitative RT-PCR experiments were carried out (Figure 7A). As described earlier^30^,^34^,^40^, incubation for 6 h was chosen to distinguish the effects due to the presence of microorganisms from those related to the trauma itself. In agreement with that observation, no induction of *Hm-myd88* expression was measured 6 h following dissection under sterile conditions. These experiments also revealed that *Hm-myd88* expression was strongly and differently modulated according to the immune challenge applied. Indeed, *Hm*-MyD88 gene expression was induced by LPS and MDP. On the contrary, it was repressed during stimulation with Gram positive bacteria, viruses and yeast. Unlike *Hm-myd88*, *Hm-sarm* was only and strongly expressed 6 h following dissection under sterile conditions (Figure 7B). Moreover, when *H. medicinalis* nerve cords were experimentally infected by various pathogens and microbial components, the up-regulation of *Hm-sarm* was abolished and quite contrarily its expression was repressed.

### Leech neuronal response to lipopolysaccharide (LPS) may involve an *Hm*-MyD88-dependent signaling pathway

**Figure 8:**
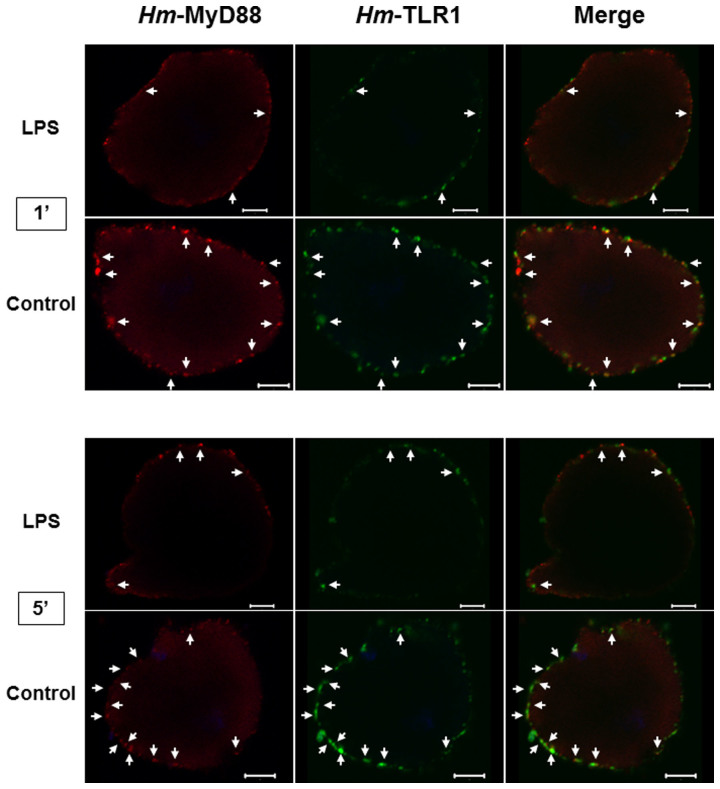
Neurons could respond to lipopolysaccharide (LPS) through a *Hm*-MyD88 dependent signaling pathway. Dissociated neurons were incubated with 100 ng/mL LPS for 1 and 5 minutes and double-labeled with anti-*Hm*-MyD88 (red) and anti-*Hm*-TLR1 (green). Untreated neurons served as controls. Arrows indicate regions where *Hm*-MyD88 and *Hm*-TLR1 show partial co-localization. Scale bar = 5 μm.

Quantitative RT-PCR results suggested that the MyD88 pathway may be conserved in leech CNS challenged with LPS. Therefore, to determine if leech neurons could indeed respond to LPS through *Hm*-MyD88, isolated neurons were challenged with LPS for 1 and 5 minutes and labelled with an anti-*Hm*-MyD88 (Figure 8). A detection of the endosomal *Hm*-TLR1^30^ was also carried out to assess the specificity of the reaction. *Hm*-TLR1 is intracellular and thus unlikely to respond directly to LPS and to launch a subsequent *Hm*-MyD88-dependent signalling pathway.

In untreated neurons, *Hm*-MyD88 were detected as aggregates in the vicinity of the cell surface and in close localization with *Hm*-TLR1 (arrows). In contrast, *Hm*-MyD88 labelling was more diffuse in neurons treated with LPS for 1 and 5 minutes. As expected, co-localization between *Hm*-MyD88 and *Hm*-TLR1 disappeared. The movement of this adaptor in such a short period of time strongly suggests its recruitment by an uncharacterized receptor involved in LPS recognition. It also suggests that the response of leech neurons to LPS may involve an *Hm*-MyD88-dependent signaling pathway.

### Muramyl dipeptide (MDP) induces a redistribution of *Hm*-TLR1 and *Hm*-MyD88 in leech isolated neurons

**Figure 9:**
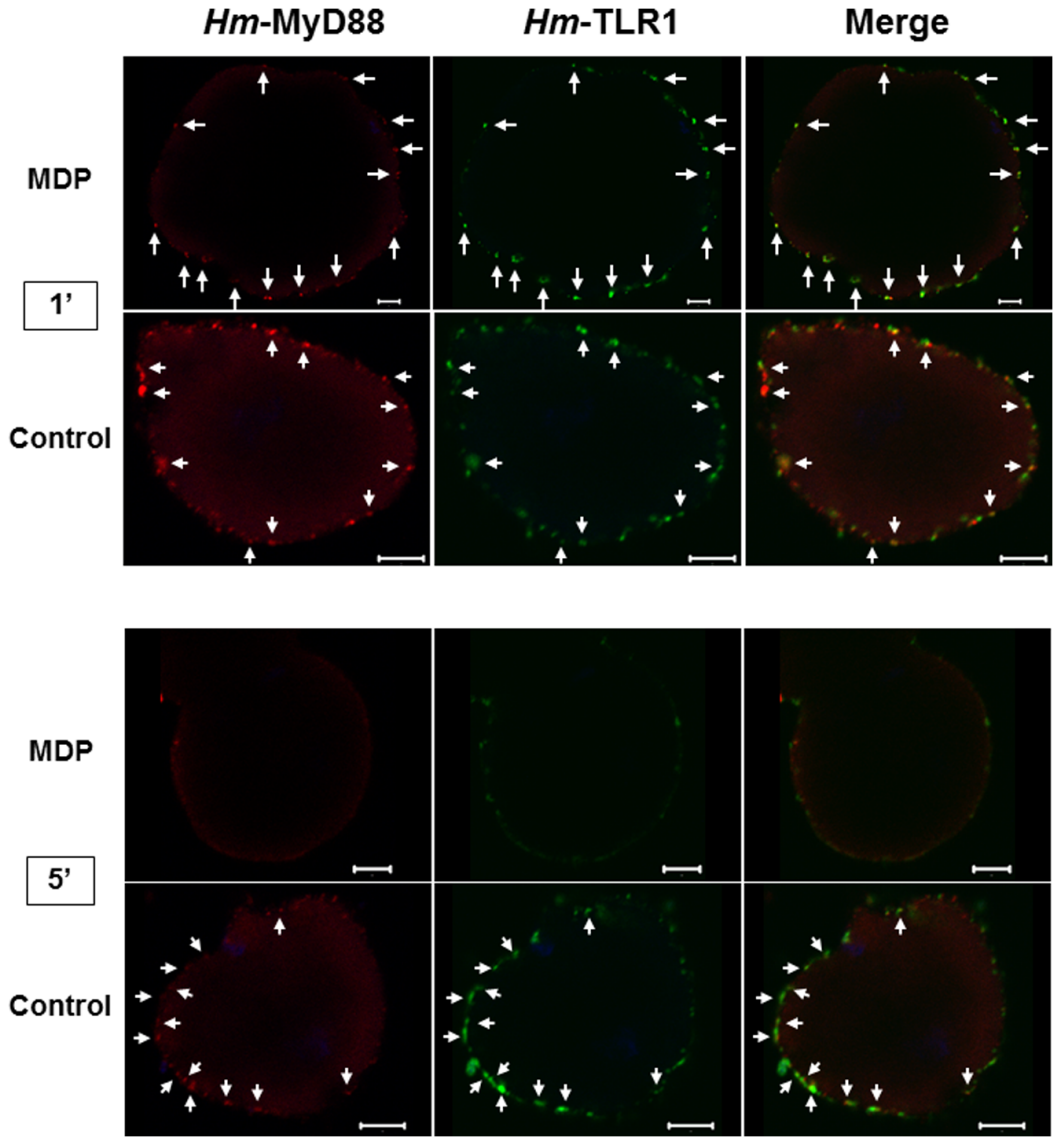
Muramyl dipeptide (MDP) induces a redistribution of *Hm*-MyD88 and *Hm*-TLR1 in neurons. Dissociated neurons were incubated with 10 μg/mL MDP for 1 and 5 minutes and double-labeled with anti-*Hm*-MyD88 (red) and anti-*Hm*-TLR1 (green). Untreated neurons served as controls. Arrows indicate regions where *Hm*-MyD88 and *Hm*-TLR1 show partial co-localization. Scale bar = 5 μm.

Several studies support the role of muramyl dipeptide (MDP) in the process of neuro-immune modulation^47^,^48^,^49^. In mammals, MDP is a ligand for the intracellular sensor NOD2, a member of the NLR family. The cooperation between NOD2 and TLR is now clearly demonstrated in various cell types including microglia^50^,^51^,^52^. Interestingly, stimulation of leech CNS with MDP induced the expression of the genes encoding *Hm*-TLR1, the cytokine *Hm*-EMAPII^40^ and *Hm*-MyD88 (Figure 7A). It is to be noted that, during a bacterial challenge, *Hm*-emapii is under the control of *Hm*-TLR1 signaling pathway. This raises the likehood that leech CNS is primed by MDP and exhibits a NLR signaling pathway. In addition, these results suggest that cooperation between NLR and TLR also exists. Thus, to test a putative MDP priming effect on leech TLR signaling pathways, isolated neurons were challenged with MDP for 1 and 5 minutes and double-labeled with anti-*Hm*-MyD88 and anti-*Hm*-TLR1 (Figure 9). The controls used were the same as those presented for the LPS challenge.

Compared with untreated neurons, *Hm*-MyD88 and *Hm*-TLR1 underwent a potent redistribution when the cells were treated with MDP. At 1′, some aggregates were still apparent for both proteins. Interestingly, significant co-localization between the receptor and the adaptor were still observed (arrows). From 1′ to 5′, the movement of *Hm*-MyD88 and *Hm*-TLR1 continued and at 5′ the two proteins were hardly detectable. This reveals that MDP priming on leech CNS affected not only delayed physiological processes (e.g. genes expression) but also early responses such as the movement of key proteins involved in TLR signalling pathways. This also supports the idea that the cooperation between leech NLR sensor and leech TLR may be a general characteristic of leech CNS immune response.

### Leech CNS repair may involve activation of *Hm*-MyD88 and *Hm*-SARM pathways

**Figure 10:**
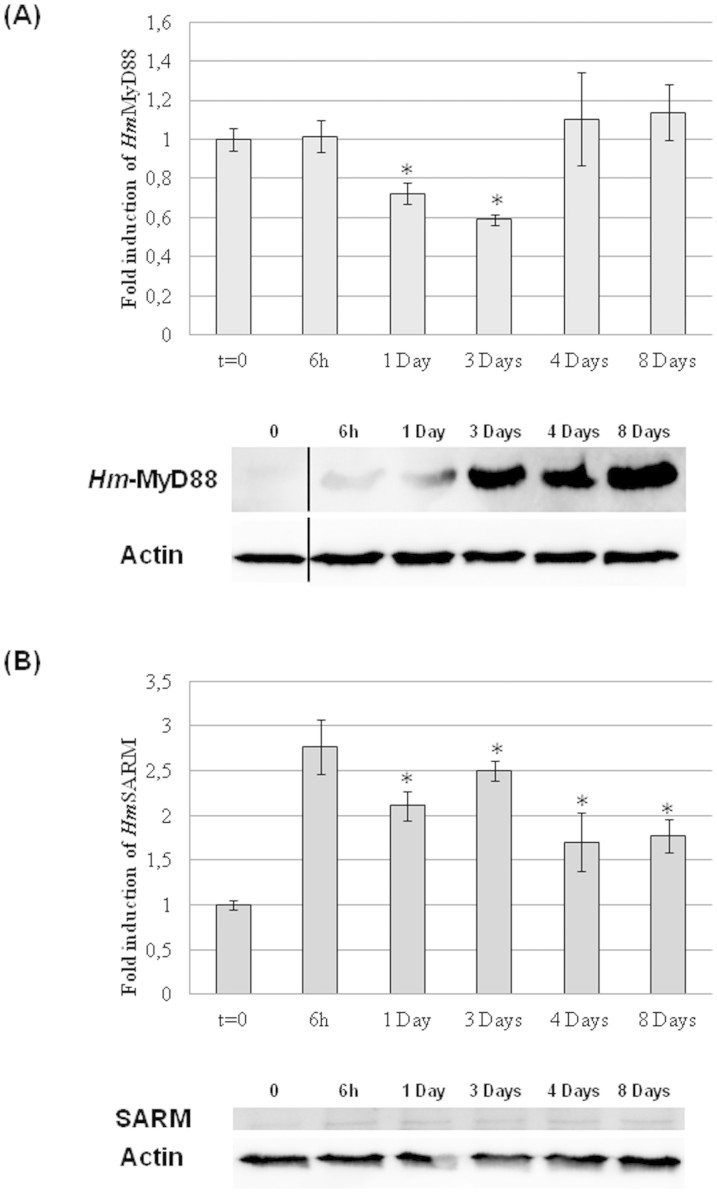
Kinetics of expression in the course of neural regeneration. (A) *Hm*-MyD88 and (B) *Hm*-SARM gene and protein expressions were assessed by qRT-PCR and western blot in isolated nerve cords cultured *ex vivo* for up to 8 days under sterile conditions. Samples were collected at 0 h (T0), 6 h, 1 day, 3 days and 8 days. qRT-PCR results are presented as graphics. Graphics represent the best results of two independent experiments that displayed similar variations; p-values from Student's T-tests were calculated between the different conditions, based on experimental measurements performed in triplicate (*p < 0.05). For western blot experiments, membranes were incubated with anti-*Hm*-MyD88 and anti-SARM. To assess that an equal amount of proteins was loaded on gels, membranes were stripped and reprobed with anti-Actin.

The medicinal leech is able to regenerate its CNS after a lesion. Our previous studies have shown that this regenerative process was enhanced when the nerve cord was also facing a microbial challenge^34^. This was linked to the release of antimicrobial peptides exhibiting neurotrophic properties. The dual role of these molecules in leech CNS immunity and repair raises the likelihood that common signaling pathways are triggered after an infection or lesion. This was confirmed by our quantitative RT-PCR and western blot experiments performed in conditions of neural regeneration (Figure 10). For this purpose, nerve cords maintained in culture for up to eight days under sterile conditions were lesioned at T0 by cutting completely through half of a connective nerve that links two adjacent ganglia. The time intervals were chosen in reference to the observations reported by Müller, who demonstrated that synaptic connections and normal functions of axotomized leech neurons were restored eight days after injury^53^. Cultures were stopped at different time post axotomy. Our real-time quantitative RT-PCR experiment showed that the level of *Hm*-MyD88 mRNA decreased progressively during the first three days of repair and returned to the basal level on the fourth day and until repair is achieved (Figure 10A). This decrease reflects the synthesis of the protein from pre-existing messengers as revealed by western blot analysis (Figure 10A). Indeed, *Hm*-MyD88 protein level increased progressively in course of regeneration and peaked when regeneration is achieved. Concerning *Hm*-SARM, we observed that both mRNA and protein levels increased as soon as six hours and remained elevated all along the repair process. Thus, *Hm-sarm* gene expression may be induced to maintain constant the amount of *Hm*-SARM protein and control CNS repair (Figure 10B).

## Discussion

In the present study, we present the identification of the first lophotrocozoan SARM. We also report for the first time the characterization of MyD88 in the CNS of an invertebrate. To the best of our knowledge, we clearly showed for the first time that differentiated neurons of the CNS could respond to LPS through a MyD88-dependent signaling pathway. Such a recruitment of a MyD88 ortholog after LPS challenge was also never demonstrated in any protostomian or non-mammalian deuterostome. In vertebrates and invertebrates, MyD88 plays a central role in the innate immune system. It is the adaptor molecule in majority of the TLRs and it transmits the signals necessary to mount an efficient immune response. Consequently, stimulation of TLR signaling by various microbial derivatives strongly modulates MyD88 gene expression^10^,^11^,^13^,^42^,^54^,^55^. Our quantitative RT-PCR experiment revealed that the expression of *Hm*-MyD88 encoding gene was also tightly regulated in leech CNS experimentally infected by various pathogens and microbial components (Figure 7A). Undeniably, its expression was induced during challenges with LPS and MDP, while it was repressed during stimulation with Gram-positive bacteria, viruses and yeast. A survey of leech genome and transcriptome databases reveals the presence of at least five TLRs and several splice variants^33^. This and the differential pattern of expression of *Hm*-Myd88 gene suggest that different PRRs are involved to detect these various microbial compounds and to launch an accurate immune response against bacteria, viruses and fungi. This immune reaction will in turn induce the differential secretion of antimicrobial peptides (AMPs) such as neuromacin and *Hm*-lumbricin^34^ as well as the release of the chemokine *Hm*-EMAPII^40^.

The observed induction of *Hm*-myd88 after LPS and MDP challenge prompted us to examine the role of this adaptor at the functional level. To achieve this study, isolated neurons were stimulated with LPS or MDP for 1 and 5 minutes and then labeled with an anti-*Hm*-MyD88 (Figure 8 and 9). We report that stimulation of leech neurons with LPS triggered a redistribution of *Hm*-MyD88 at the cell surface. This suggests its recruitment by a receptor involved in LPS recognition. It shows for the first time that a non-mammalian CNS could respond to LPS through a MyD88-dependent signaling pathway. In mammals, the effect of LPS challenge on CNS has been tested extensively. However, studies describing the direct effect of LPS on neurons and the outcomes of such treatment are scarce and controversial. Indeed, some works demonstrated that neurons were unresponsive to this PAMP^56^,^57^, while other studies depicted their possible involvement as key sensors of LPS to launch CNS inflammation^58^,^59^,^60^. Besides, whereas LPS treatment on cortical neurons caused their fragility^61^, survival of P60 DRG neurons was not affected^58^. Moreover, signals mediated by TLR4 in neurons are not well understood and were depicted as non-canonical for a long time^62^. Nevertheless, it was shown that LPS activated NF-κB in chemosensory neurons from NPJgc^63^ and P60 DRG neurons^58^, suggesting the involvement of MyD88-dependent mechanisms. To the best of our knowledge, our study is the first to demonstrate directly that differentiated neurons of the CNS could respond to LPS through a MyD88-dependent signaling pathway. It gives therefore an important clue regarding molecular mechanisms underlying LPS response in differentiated neurons of the CNS. Keeping in mind that the medicinal leech is able to regenerate its CNS and that chemokines favour this repair^31^, LPS treatment on leech neurons may therefore contribute to a better understanding of the effect of this PAMP on axon growth, neuron survival and inflammatory response.

**Figure 11:**
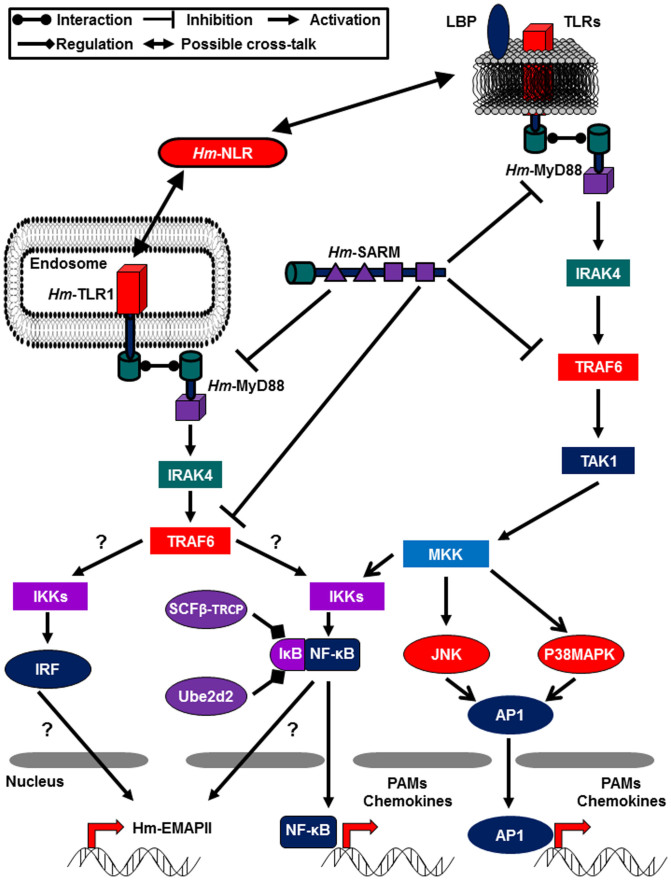
The putative TLR4-like and *Hm*-TLR1 pathways in leech neurons. The survey of *H. medicinalis* databases and our experiments^30^,^33^ pointed out that leech possesses (i) the main components of the canonical TLR4/LPS pathway and (ii) the molecules recruited by endosomal TLRs. “?” indicates that stimulation of *Hm*-TLR1 may activate an IRF or a NF-κB signaling.

In mammalian CNS, the response to LPS is mediated by TLR4 expressed at the cell surface of astrocytes^64^, microglia^65^, and neurons^60^. Analysis of leech genome and transcriptome databases^33^ reveals the presence of key molecules implicated in LPS recognition and signalling in mammals, *i.e.* a TLR closely related to mammalian TLR4, several LBP/BPI, *Hm*-MyD88, Traf6, IκBα, and two IκBα regulators: the E3 ubiquitin ligase SCF^β-TrCP^ and the E2 ubiquitin conjugating enzyme Ube2d2^3^,^66^,^67^. This suggests that a true TLR4-like pathway could exist in leech (Figure 11). Indeed, apart from mammals and birds^68^, where the LPS receptor is clearly a TLR, it signals through a TLR-independent pathway in other species^9^,^10^,^69^,^70^,^71^. For example, in sponge, LPS is bound by the specific poriferan molecule SLIP to trigger a MyD88-dependent signaling pathway^10^. The existence of alternative receptors has also been evocated in the horseshoe crab^72^ and fish^73^ *i.e.* factor C and beta-2 integrins, respectively.

Data obtained with Muramyl dipeptide (MDP) further suggests the similarity of the leech PRR pathways to its mammalian counterpart. In vertebrate CNS, MDP, the ligand of NOD2, is a potent immune modulator^47^,^48^,^49^ and exerts priming and synergistic effects for TLR ligand activities^50^,^51^,^52^,^74^. In this line, in human monocytic cells in culture, MDP also up-regulated MyD88 mRNA expression^74^. Similarly, we showed that stimulation of leech CNS with MDP induced the expression of the genes encoding *Hm*-TLR1^30^, the cytokine *Hm*-EMAPII^40^ and *Hm*-MyD88 (Figure 7A). Our immunofluorescence experiment also revealed that a stimulation of leech neurons with MDP triggered a potent redistribution of *Hm*-MyD88 and *Hm*-TLR1 in the cells (Figure 9). It is the first time that such movements of a TLR and MyD88 are demonstrated in neurons. This raises the likehood that leech CNS is primed by MDP, exhibits a NLR signaling pathway and that cooperation between NLR and TLR exists (Figure 11). Despite the fact that the leech receptor for MDP is still unknown, the recent characterization of *Hm*-NLR, a NLR-like sensor ortholog, and its common pattern of expression with *Hm*-TLR1 in neurons support this hypothesis^30^. This also supports the idea that cooperation between leech NLR sensor and leech TLR may be a general characteristic of leech CNS immune response. However, in leech injured CNS, MDP blocked microglia recruitment to the lesion site^29^. Thus, MDP may exert a dual effect on leech CNS: (i) prime neurons to ensure an efficient immune response and (ii) control the amount of microglia attracted to avoid an over inflammation deleterious for the nervous system.

Another key mechanism to balance the inflammatory response and avoid an immune signalling storm is to shut down TLR pathways^75^. Among negative regulators of TLR signalling, SARM is the most conserved across evolution. Indeed, in mammals^18^,^23^,^24^; protochordates^19^,^25^ and arthropods^16^,^26^, SARM inhibits the TLR signaling pathways specifically via MyD88 and/or TRIF and/or TRAF6. To control this precise switch between pathway activation and repression, the time-course of SARM expression is tightly regulated in immune cells during an immune challenge^16^,^18^,^19^,^23^,^24^,^26^. Our quantitative RT-PCR experiment revealed that the levels of *Hm*-SARM messengers significantly diminished in leech nerve cords experimentally infected during 6 hours by various pathogens and microbial components (Figure 7B). At this time point, we have previously demonstrated that *H. medicinalis* CNS released beneficial immune factors to mount an immune response and restore homeostasis^29^. Therefore, we may expect *Hm*-SARM to be synthesized from a pre-existing pool of messengers to slow down TLR pathways by its interaction with *Hm*-MyD88 and/or *Hm*-TRAF6^33^. This may adjust the amount of AMPs and chemokines secreted. However, to prevent *Hm*-SARM from abolishing TLRs signalling, its encoding gene is repressed to avoid a subsequent synthesis of the protein. On the contrary, we can't exclude that *Hm*-SARM is required to initiate an innate immune response in leech CNS infected with various pathogens as it was observed in *C. elegans* challenged with bacteria and fungi^15^,^27^ and during a viral infection of mammalian brain^28^,^76^. Thus, the down-regulation of leech *Hm*-SARM gene may suppress excessive innate immunity and prevent tissue damage. Indeed, in brains of mice experimentally infected with vesicular stomatitis virus, an excessive inflammatory response involving SARM signaling led to neuronal injury^76^.

Moreover, the present study demonstrated that a tight link between CNS repair and neuroimmunity exists in this lophotrochozoan model. We showed that the amount of *Hm*-MyD88 mRNA decreased progressively in course of regeneration while the protein level increased and peaked when regeneration is achieved (Figure 10A). *Hm*-MyD88 pathway may favour the regeneration by stimulating the synthesis of antimicrobial peptides as well as chemokines like *Hm*-EMAPII known to be critical for regeneration processes^29^,^31^,^32^,^34^,^40^,^77^. Concerning *Hm*-SARM, we observed that its gene is induced all along the repair process in order to sustain the protein synthesis (Figure 10B). In mammals, SARM regulates the morphology of hippocampal neurons^78^ and controls their death during metabolic stress^20^. It also activates an injury-induced axon death pathway known as Wallerian degeneration in mice and drosophila^79^. In leech, isolated neurons such as Retzius neurons and anterior pagoda neurons start sprouting a few minutes after being plated^80^ and neurite growth is not random^81^. In accordance with the literature mentioned above, we may expect *Hm*-SARM to control the complex dynamics of neurite elongation and retraction as well as the elimination of misdirected sprouts observed during axon growth^80^,^82^.

Taken together, our data revealed that *Hm*-MyD88 and *Hm*-SARM pathways may fully contribute to set a balance between stimulatory and inhibitory signals in leech to ensure CNS repair and defense.

## Accession codes:

*Hm*-MyD88 mRNA, *Hm*-SARM mRNA, *Hm*-myd88 gene and *Hm*-sarm gene sequences were deposited in the NCBI GenBank under accession no. KM233119; KM233120; KM233121 and KM233122 respectively.

## Supplementary Material

Supplementary Information Supplementary Information
