# Supplementary material for: Hm-MyD88 and Hm-SARM: Two key regulators of the neuroimmune system and neural repair in the medicinal leech
Source: Sci Rep. 2015 Apr 16;5:9624. doi: 10.1038/srep09624 (PMC4399414; doi:10.1038/srep09624)
Supplement: Supplementary Information [file srep09624-s1.pdf]

Hm-MyD88 and *Hm*-SARM: Two key regulators of the neuroimmune system and neural repair in the medicinal leech.

Rodet F., Tasiemski A., Boidin-Wichlacz C., Van Camp C., Vuillaume C., Slomianny C., Salzet M.

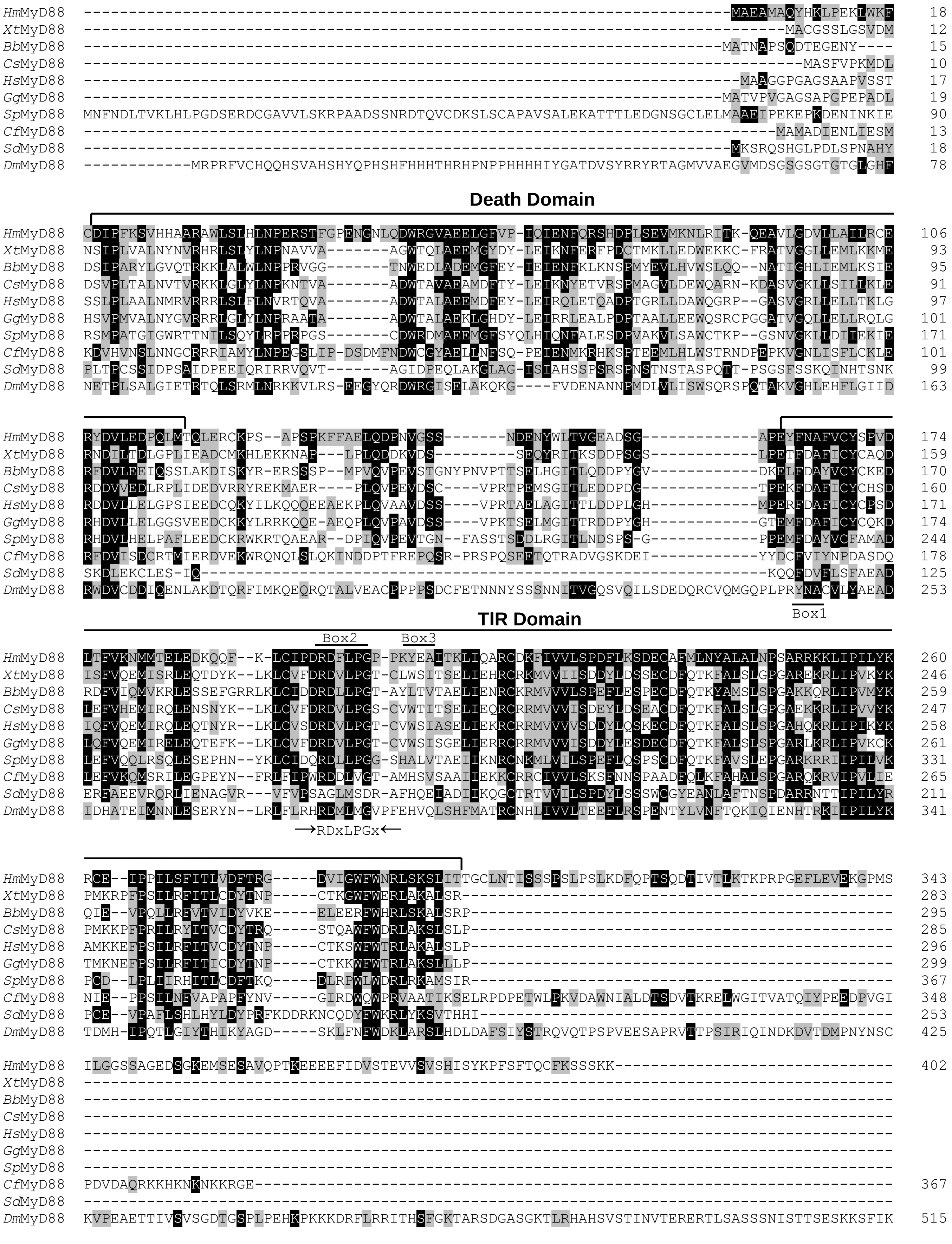

		% Identity	% Similarity
*Hm*MyD88	---------------------	-	-
*Xt*MyD88	----------------------	36	53
*Bb*MyD88	----------------------	34	52
*Cs*MyD88	----------------------	33	53
*Hs*MyD88	----------------------	32	51
*Gg*MyD88	----------------------	32	50
*Sp*MyD88	----------------------	26	42
*Cf*MyD88	----------------------	23	42
*Sd*MyD88	----------------------	21	41
*Dm*MyD88	WQPNILKKALFSRSSSKLQTPG 537	18	37

<u>Supplementary Material 1:</u> Comparison of the amino acid sequence of *Hirudo medicinalis* MyD88 (*Hm*MyD88) with vertebrate and invertebrate MyD88 orthologs.

Alignment was generated by using the program CLUSTAL W. Identical residues with *Hm*-MyD88 are shaded in black, homologous residues in grey. Gaps (-) were introduced to optimize sequence alignment. →RDxLPGx← indicates the key motif for MyD88 dimerization and its interaction with TLRs. Accession numbers in EMBL/Genbank: *Xt*MyD88: *Xenopus tropicalis* (NP_001016837); *Bb*MyD88: *Branchiostoma belcheri* (ABQ32299); *Cs*MyD88: *Cynoglossus semilaevis* (ACU31062.1); *Hs*MyD88: *Homo sapiens* (Q99836.1); *Gg*MyD88: *Gallus gallus* (A5HNF6.1); *Sp*MyD88: *Strongylocentrotus purpuratus* (XP_780590); *Cf*MyD88: *Chlamys farreri* (ABB76627); *Sd*MyD88: *Suberites domuncula* (Q4W1E7); *Dm*MyD88: *Drosophila melanogaster* (NP_610479).

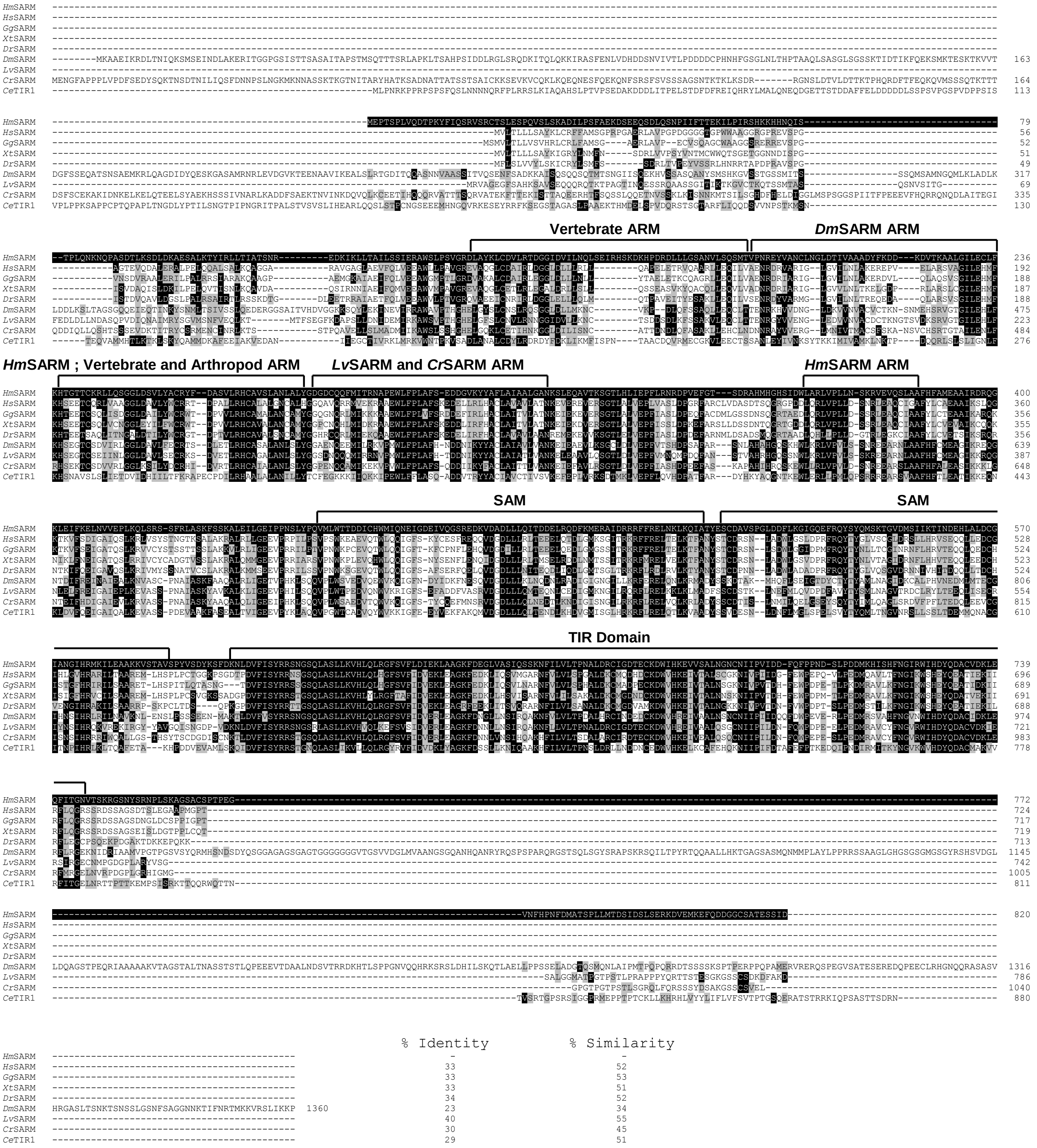

<u>Supplementary Material 2:</u> Comparison of the amino acid sequence of *Hirudo medicinalis* SARM (*Hm*-SARM) with vertebrate and invertebrate SARM orthologs.

Alignment was generated by using the program CLUSTAL W. Identical residues with *Hm*SARM are shaded in black, homologous residues in grey. Gaps (-) were introduced to optimize sequence alignment. Accession numbers in EMBL/Genbank: *Hs*SARM: *Homo sapiens* (Q6SZW1); *Gg*SARM: *Gallus gallus* (A5HNF6.1); *Xt*SARM: *Xenopus tropicalis (*XM_002937143.2); *Dr*SARM: *Danio rerio* (B3DK97); *Dm*SARM: *Drosophila melanogaster* (Q6IDD9); *Lv*SARM: *Litopenaeus vannamei* (**G8GV23);** *Cr*SARM: *Carcinoscorpius rotundicauda* (A9X3T4); *Ce*TIR-1: *Caenorhabditis elegans* (Q86DA5).

H. medicinalis myd88			
**Intron**	**5'-Donor**	**3'-Donor**	**Intron phase**
**1**	G gtatgtg<br>Glu	ttttcag AA<br>Glu	1
**2**	G gtttaac<br>Val	aattaag TT<br>Val	1
**3**	G gtgcgta<br>Ala	tttttag CT<br>Ala	1
**4**	AG gtaggtc<br>Arg	ttatcag G<br>Arg	2
**5**	A gtaagtc<br>Ser	tatttag GT<br>Ser	1

Deuterostoma
Vertebrates

H. sapiens myd88				
**Intron**	**5'-Donor**	**3'-Donor**	**Intron phase**	***H. medicinalis* I/E junctions**
**1**	G gtgagga<br>Glu	cccacag AG<br>Glu	1	
**2**	G gtaaggg<br>Gly	cacccag GG<br>Gly	1*	i3
**3**	AG gttggct<br>Arg	cccctag G<br>Arg	2*	i4
**4**	G gtaagct<br>Gly	cttgcag GT<br>Gly	1*	i5

D. rerio myd88				
**Intron**	**5'-Donor**	**3'-Donor**	**Intron phase**	***H. medicinalis* I/E junctions**
**1**	G gtacgtc<br>Asp	tgcacag AT<br>Asp	1*	i1
**2**	G gtcagtg<br>Gly	tcaacag GA<br>Gly	1*	i3
**3**	AG gcatgtt<br>Arg	ttcttag G<br>Arg	2*	i4
**4**	G gtacaaa<br>Gly	tcttcag GA<br>Gly	1*	i5

G. gallus myd88				
**Intron**	**5'-Donor**	**3'-Donor**	**Intron phase**	***H. medicinalis* I/E junctions**
**1**	G gtgagcc<br>Glu	tttgcag AG<br>Glu	1	
**2**	G gtgagtg<br>Gly	cttacag GG<br>Gly	1*	i3
**3**	AG gtgagtg<br>Arg	tctgaag G<br>Arg	2*	i4
**4**	G gtaagat<br>Gly	ctttcag GG<br>Gly	1*	i5

X. tropicalis myd88				
**Intron**	**5'-Donor**	**3'-Donor**	**Intron phase**	***H. medicinalis* I/E junctions**
**1**	G gtaagta<br>Glu	gttacag AG<br>Glu	1*	i1
**2**	G gtacgcc<br>Gly	cttacag GG<br>Gly	1*	i3
**3**	CG gtaaggg<br>Arg	gttatag G<br>Arg	2*	i4
**4**	G gtaagat<br>Gly	cttacag GG<br>Gly	1*	i5

Protostoma
Ecdyzozoa

D. melanogaster myd88				
**Intron**	**5'-Donor**	**3'-Donor**	**Intron phase**	***H. medicinalis* I/E junctions**
**1**	G gtaggca<br>Ala	tcaaaag CC<br>Ala	1	
**2**	AGG gtgagtg<br>Arg	cttacag CTA<br>Leu	3	
**3**	G gtgagta<br>Glu	tcccag AG<br>Glu	1*	i4
**4**	AC gtaagtg<br>Thr	tatgcag T<br>Thr	1	

L. vannamei myd88				
**Intron**	**5'-Donor**	**3'-Donor**	**Intron phase**	***H. medicinalis* I/E junctions**
**1**	AG gtgaata<br>Arg	tttacag A<br>Arg	2	
**2**	T gtaatat<br>Tyr	tttacag AT<br>Tyr	1 *	i1
**3**	G gtaagga<br>Asp	attgcag AT<br>Asp	1*	i3
**4**	AAG gtaagt<br>Lys	cttgcag CTG<br>Leu	3	
**5**	AG gtaacgt<br>Ser	ctttcag C<br>Ser	2	
**6**	GAT gtaagt(<br>Asp	tcagcag TCT<br>Ser	3	

<u>Supplementary Material 3:</u> Intron-exon boundaries of the leech, vertebrates and ecdyzozoa *myd88* genes.
The sequence of each of the intron-exon boundaries is shown, as well as the codons for the amino acid residues. Uppercase and lowercase letters represent nucleotides in the exons and introns, respectively. Asterisks (*) indicate that the position of an intron-exon junction is the same in leech and a vertebrate or an ecdyzozoa gene. The column entitled "*H. medicinalis* I/E junctions" point out which leech intron-exon junction is conserved in vertebrate or ecdyzozoa genes.

H. Medicinalis sarm			
Intron	5'-Donor	3'-Donor	Intron phase
1	CAG gtatcac	gtttcag GGT	-
2	GAG gttcgaa<br>Glu	aattcag ATT<br>Ile	3*
3	CAG gtgatat<br>Gln	attttag AAT<br>Asn	3
4	AG gtctgtg<br>Arg	gttttag A<br>Arg	2*
5	A gttggca<br>Lys	cttttag AG<br>Lys	1*
6	AG gtgaatg<br>Ser	atcaaag T<br>Ser	2*
7	AAG gtaaaat<br>Lys	tttccag GAG<br>Glu	3*
8	AG gttcatg<br>Arg	ggtgcag A<br>Arg	2*

H. Sapiens sarm			
Intron	5'-Donor	3'-Donor	Intron phase
1	CG gtgagcg<br>Arg	tgggcag A<br>Arg	2
2	AAG gtgggtg<br>Lys	tccctag GTG<br>Val	3 *
3	CGG gtagagt<br>Arg	cccccag GAG<br>Glu	3
4	AG gtacgga<br>Arg	ggtgcag G<br>Arg	2*
5	G gtcagcc<br>Glu	acttcag AA<br>Glu	1*
6	AG gtgagga<br>Ser	cttgcag T<br>Ser	2*
7	AAG gtaggtg<br>Lys	tctccag GAG<br>Glu	3*
8	AA gtgagcc<br>Lys	cccacag G<br>Lys	2*

<u>Supplementary Material 4:</u> Intron-exon boundaries of the leech and human *sarm* genes. The sequence of each of the intron-exon boundaries is shown, as well as the codons for the amino acid residues. Uppercase and lowercase letters represent nucleotides in the exons and introns, respectively. Asterisks (*) indicate that the position of an intron-exon junction is the same in leech and human genes and has the same intron phasing.

***Hm*-MyD88**

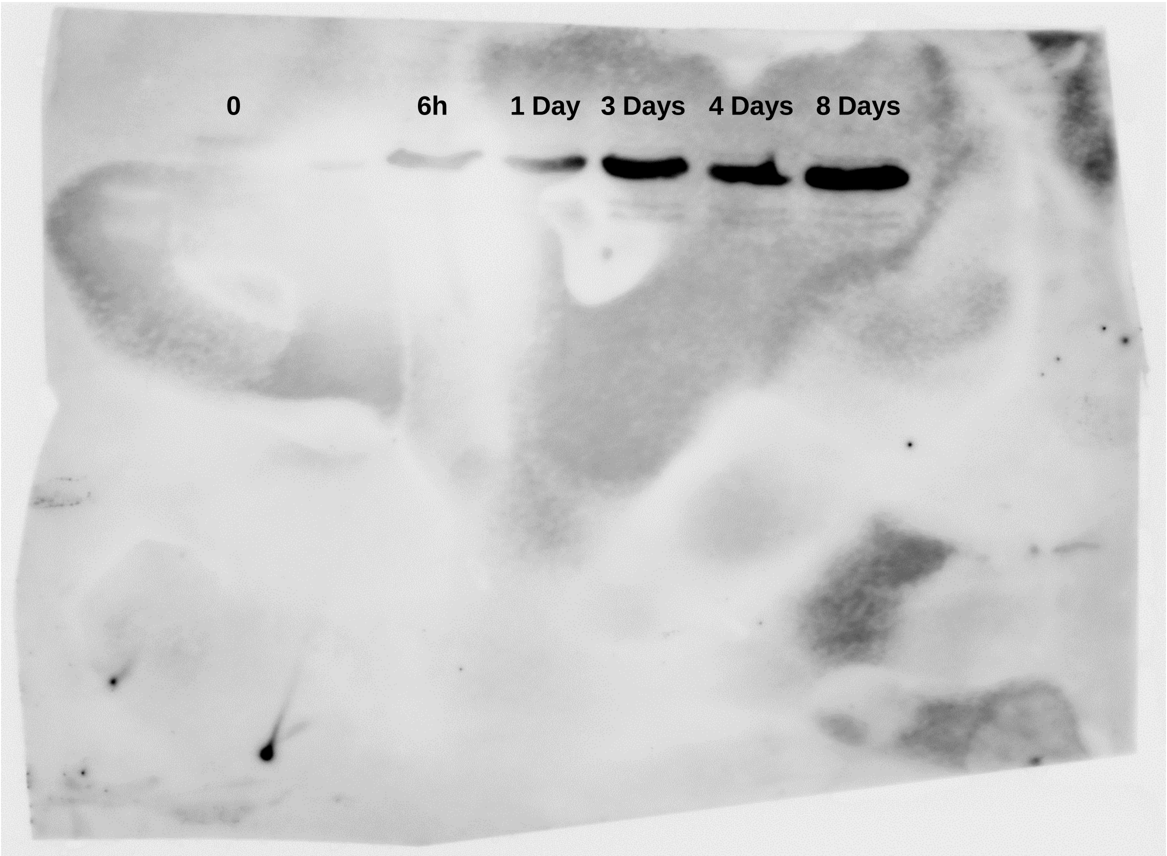

Actin

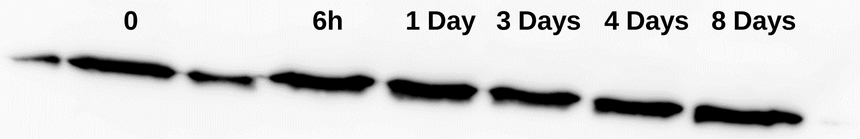

<u>Supplementary Material 5:</u> Unprocessed original scans for the blots in figure 10 (A) Kinetics of expression in the course of neural regeneration.

Hm-MyD88 protein expression was assessed by western blot in isolated nerve cords cultured *ex vivo* for up to 8 days under sterile conditions. Samples were collected at 0 h (T0), 6 h, 1 day, 3 days and 8 days. Membrane was incubated with anti-*Hm*-MyD88. To assess that an equal amount of proteins was loaded on gels, membrane was stripped and reprobed with anti-Actin.

.

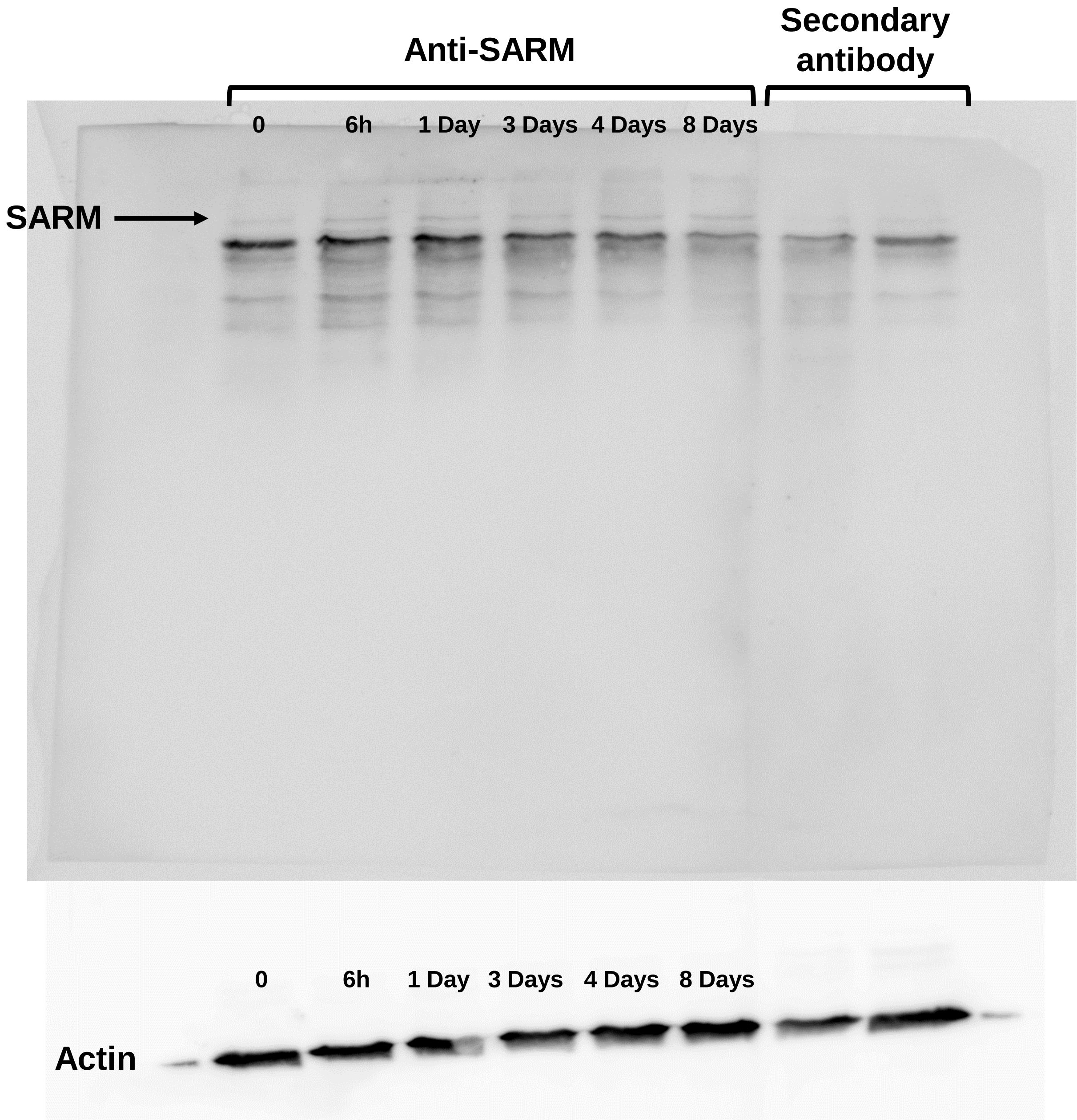

<u>Supplementary Material 6:</u> Unprocessed original scans for the blots in figure 10 (B) Kinetics of expression in the course of neural regeneration.

Hm-SARM protein expression was assessed by western blot in isolated nerve cords cultured *ex vivo* for up to 8 days under sterile conditions. Samples were collected at 0 h (T0), 6 h, 1 day, 3 days and 8 days. Membrane was incubated with anti-SARM. As a control, a membrane was incubated with the secondary antibody alone. To assess that an equal amount of proteins was loaded on gels, membrane was stripped and reprobed with anti-Actin.
